# Efficiency of Recurrent Genomic Selection in Panmictic Populations

**DOI:** 10.3390/ani15192925

**Published:** 2025-10-09

**Authors:** José Marcelo Soriano Viana, Jean Paulo Aparecido da Silva, Paulo Sávio Lopes

**Affiliations:** 1 Department of General Biology, Federal University of Viçosa, Viçosa 36570-900, MG, Brazil; jean.p.silva@ufv.br; 2 Department of Animal Science, Federal University of Viçosa, Viçosa 36570-900, MG, Brazil; plopes@ufv.br

**Keywords:** genomic prediction, realized genetic gain, prediction accuracy, inbreeding, epistasis

## Abstract

Simulation-based studies can support breeders’ decisions inexpensively, since there is no need to perform a new procedure. The objective was to assess the efficiency of recurrent genomic selection in panmictic population under additive–dominance and additive–dominance with epistasis models. We assumed two broiler chicken populations with contrasting linkage disequilibrium (LD) levels, 38,500 SNPs, and 1000 genes controlling feed conversion ratio. We applied recurrent genomic selection over seven cycles. The genomic selection efficacy, expressed as realized total genetic gain, was proportional to the LD level and genotypic variance. Genomic selection required model updating to achieve a higher efficacy. The training set size required by genomic selection can be as low as 10%/generation. Under this low-cost scenario, the genomic selection efficacy was slightly lower than the maximum efficacy. There is no difference between genetic evaluation methods regarding the decrease in the genotypic variance due to selection. In general, additive value prediction accuracies and realized genetic gains were highly correlated. The accumulated inbreeding level was not high due to avoidance of sib cross. The genomic inbreeding coefficient over generations was close to zero. Except for dominant epistasis, the efficacy of genomic selection was 4.1 to 46.2% lower than the efficacy under no epistasis.

## Simple Summary

Simulation-based studies offer knowledge that can only be indirectly provided by empirical investigations. The objective of this simulation-based study was to assess the efficiency of recurrent genomic selection in a panmictic population under additive–dominance and additive–dominance with epistasis models. Genomic selection efficacy is proportional to the population linkage disequilibrium and genotypic variance. Genomic selection efficacy can be increased by model updating. Genomic selection efficacy can be kept at a reasonable level, with a minimum of 10% of genotyped and phenotyped individuals. Non-allelic gene interaction decreases genomic selection efficacy. The inbreeding that occurs over generations of genomic selection should be minimized by avoiding sub-crosses. We demonstrated that genomic selection is an efficient method for animal breeding, especially for complex traits.

## 1. Introduction

Considering simulation-based and empirical studies on genomic selection/genomic prediction of complex traits, human geneticists, as well as animal and plant breeders, recognize the significance of genotyping and phenotyping a limited number of individuals in populations, aiming to predict the genomic status of non-phenotyped individuals who are genotyped. The application of genomic selection in dairy cattle increased the rate of genetic improvement in the range of 50–100% for most traits. The increases were even higher for some low-heritability traits [1]. Jones and Wilson [1] consider that genomic selection significantly decreases the time for accurate breeding value prediction, for a fraction of the cost of traditional progeny testing. Genotyping is expensive, but the cost has decreased over the years. Genotyping is now cost-effective in some livestock species, particularly dairy cattle, because genomic selection reduces progeny testing costs and generation interval, whereas in small-bodied species like poultry, the cost–benefit ratio depends on using lower-cost genotyping strategies (e.g., low-density panels with imputation or low-pass sequencing). Some concerns in long-term use of genomic selection are a fast decrease in the additive variance [2] and a rapid increase in the inbreeding level [3].

In recent years, simulation-based investigations have provided additional evidence that genomic selection is an effective genetic assessment process for livestock species. These studies also investigated the impact of short- and long-term genomic selection on the genetic variability and inbreeding level. Zhao et al. [4] employed a simulated and an empirical pig dataset to investigate genomic mating optimization over five selection cycles. There was discrimination among the seven mating schemes only for the trait heritability of 0.5. Regardless of the trait heritability, the higher increase in the inbreeding coefficient (F) occurred with positive assortment of parents. Zheng et al. [5] assessed the long-term impact of genomic selection in a simulated beef cattle population. They observed no influence of SNP density on genetic gain or inbreeding level. The genetic gains were proportional to the trait heritability. Genomic best linear unbiased prediction (GBLUP) and BayesA provided similar genetic gains, which were superior to the gains provided by phenotypic selection. The increase in the inbreeding coefficient was higher for GBLUP and lower for phenotypic selection. Chuang et al. [6] simulated two dual-purpose chicken breeding programs and assessed the genetic gains with genomic selection over 40 generations, including or not including gene editing. The genetic gain was proportional to the population genotypic variance and the number of edited genes. Gene editing accelerated the decrease in additive variance.

Wientjes et al. [7] also assessed the long-term effects of genomic selection on a livestock simulated population. Regardless of the genetic architecture, they observed decreases in the additive value prediction accuracy and the additive genetic variance. Epistasis negatively affected the accumulated genetic gains. The authors concluded that genomic selection outperforms pedigree selection in terms of long-term genetic gain but results in a similar reduction in genetic variance. Thomasen et al. [8] simulated five dairy cattle breeding programs to compare genomic selection with conventional progeny testing. The genetic gains/year were three to four times higher with genomic selection, depending on linkage disequilibrium (LD) and population size. The increase in the inbreeding coefficient was lower with genomic selection, especially in the small reference population. Using simulated populations of purebred swine, Li et al. [9] investigated the relative importance of genotypic and phenotypic information on the additive value of genomic prediction accuracy based on single-step (ss) GBLUP. They found that a genotyping rate of 40–50% for each litter in three to four previous generations, depending on the trait heritability, can provide yield prediction accuracy comparable to that of a genotyping rate of 100%.

The empirical investigations are generally in full agreement with the simulation-based studies regarding genetic gain and inbreeding. Applying genomic selection over three generations in a paternal chicken line, Tan et al. [10] observed significant increments in body weight (over 20%) and meat production (over 30%). Using a population of Rendena cattle, Mancin et al. [11] observed that ssGBLUP and weighted ssGBLUP presented higher accuracy and reliability than pedigree-based BLUP. Lozada-Soto et al. [12] assessed changes in genetic diversity and inbreeding in an American Angus cattle population under genomic selection for 17 years. In general, genetic diversity was conserved, and the pedigree and genomic inbreeding accumulation rates decreased. They also observed that the inbreeding depression for growth traits was more affected by the genomic than by the pedigree-based inbreeding coefficient.

Hollifield et al. [13] assessed the decay in the additive value prediction accuracy over nine generations of commercial pig populations. The average decay in accuracy from the first generation after training to generation 9 was −19 and −41% for the growth and fitness traits, respectively. Makanjuola et al. [14] also investigated the effect of long-term genomic selection on the rate of inbreeding in Holstein and Jersey cattle populations. They observed increases/generation in the range 1.2 to 2.1%. Cuyabano et al. [15] assessed the additive value prediction accuracy across five generations in two Korean pig breeds. They concluded that it is necessary to continuously update the reference population with new genotypes and phenotypes. However, it may not be necessary to keep all ancestral genotypes indefinitely in the reference population.

Considering that there are few simulation-based studies on the efficiency of recurrent genomic selection, including epistatic effects, most with no specification of the epistasis type, our objective was to assess the efficacy of recurrent genomic selection in panmictic populations with contrasting LD levels, assuming additive, dominance, and epistatic effects, and defining seven types of digenic epistasis.

## 2. Materials and Methods

### 2.1. Modeling

We used the software REALbreeding (available upon request) for simulating the genome, the individuals in the two broiler chicken populations over selection cycles, and the trait. The software has GUI and versions for Windows, Linux, and macOS. It has been used for studies involving genomic selection [16], GWAS [17], QTL mapping [18], and quantitative genetics [19]. The software uses inputs from the user to simulate individual genotypes, including genes and markers, as well as phenotypes, in three stages: genome simulation, population simulation, and trait simulation.

In the first step, the user specifies the chromosome number, the number and density of markers by chromosome, the number of genes (QTLs—major genes and minor genes) by chromosome, and the trait number. To include epistasis, the user should inform the type and the number of epistatic genes. In the second step, the user specifies the population, progeny number and size, generation number, and simulation number (including resamplings of the population). The user can define full-sibs, half-sibs, or selfed progeny. In the last step, the user specifies each trait. The information required includes maximum and minimum genotypic values for homozygotes (ignoring epistasis), maximum and minimum phenotypic values (to avoid outliers), degree and direction of dominance, and broad-sense heritability. The additive, dominance, and epistatic genetic values, general and specific combining ability effects, or genotypic values, depending on the population, are computed using the following parameters: a (difference between the genotypic value of the superior homozygote and the average of the genotypic values for the homozygotes—m) and d (dominance deviation) for genes; the epistatic effects for pairs of interacting genes; the allele frequencies; and the LD values.

The parameters m and a for genes are computed from the maximum and minimum genotypic values for homozygotes. The parameter d for genes is computed from the degree and direction of dominance. For each pair of interacting genes, there are nine epistatic effects, one for each genotype, but only two to four genotypic values, depending on the digenic epistasis type. For example, the genotypic value of individual AABB is G22 = m1 + m2 + a1 + a2 + I22, where I is the epistatic effect. To obtain a solution, the software samples a random value for I22 from a probability distribution and computes the other eight epistatic effects (I21, I20, …, and I00), considering the epistasis type. For example, assuming complementary epistasis, the solution satisfies G22 = G21 = G12 = G11 and G20 = G10 = G02 = G01 = G00. The software assumes $\sigma_{I22}^{2}\sim N\left(0,k\left(\sigma_{A}^{2}+\sigma_{D}^{2}+2\sigma_{A,D}\right)\right)$, where k is a positive value defined by the user and $\sigma_{A}^{2}$, $\sigma_{D}^{2}$, and $\sigma_{A,D}$ are the additive and dominance variances and covariance, relative to the two epistatic genes. Then, the epistatic genetic values—additive x additive (AxA), additive x dominance (AxD), dominance x additive (DxA), and dominance x dominance (DxD)—are computed based on Kempthorne [20].

The allele frequencies for markers and genes are generated from beta distribution. The phenotypic values are computed from the genotypic values, assuming error effects sampled from a normal distribution. The reference population is created by crossing two populations in linkage equilibrium, followed by a generation of random crosses to achieve Hardy–Weinberg equilibrium. In this population (generation 0), the LD value in the gametic pool of generation −1 is ${∆}_{ab}^{\left(-1\right)}=P\left(AB\right).P\left(ab\right)-P\left(Ab\right).P\left(aB\right)=\;\left[\left(1-2\theta_{ab}\right)/4\right]\left(p_{a1}-p_{a2}\right)\left(p_{b1}-p_{b2}\right)$, where θ is the recombination rate, p is the allele frequency, and the indexes 1 and 2 refer to the parental populations.

### 2.2. Study Design

In the first step, we defined a genome of 10 chromosomes with variable lengths, ranging from 199.4 (chromosome 1) to 25.7 (chromosome 10) cM. In these chromosomes, we distributed 38,500 single-nucleotide polymorphisms (SNPs), from 10,000 (chromosome 1) to 1250 (chromosome 10), and 1000 genes, from 260 (chromosome 1) to 30 (chromosome 10), at random positions using a uniform distribution. The average density for SNPs and genes was 0.02 and 0.77 cM, respectively. The number of SNPs is compatible with the Affymetrix 55K SNP chip. In the second step, we simulated two populations with contrasting LD levels, using the same genome. In both populations, the average MAF for SNPs was 0.3, and the average frequency for the favorable recessive alleles was 0.6. The low LD population was generated from populations with MAFs of 0.3 and 0.3 for SNPs and favorable allele average frequencies of 0.6 and 0.6. The high LD population was generated from populations with a MAF of 0.1 and 0.5 for SNPs and favorable allele average frequencies of 0.3 and 0.9. The initial sample size was 400 females and 400 males. In the third step, we simulated the feed conversion ratio (FCR). To allow computing the genetic parameters m, a, and d, we defined 1.1 and 2.9 as the minimum and maximum genotypic values for homozygotes. We defined 1.0 and 3.5 as the minimum and maximum phenotypic values. We also assumed positive dominance. The degree of dominance ranged from 0.0 to 1.2 (0.6 on average). The broad sense of heritability was 30% across generations. We kept the parameters a and d for each gene for both populations. We initially assumed only additive and dominance effects. Then, we assumed digenic epistasis for 50% of the genes in the high LD population. In this case, we assumed complementary (9:7 in F_2_), duplicate (15:1 in F_2_), dominant (12:3:1 in F_2_), recessive (9:3:4 in F_2_), and dominant and recessive (13:3 in F_2_) epistasis, duplicate genes with cumulative effects (9:6:1 in F_2_), and non-epistatic gene interaction (9:3:3:4 in F_2_). We also assumed all types of epistasis. In this case, REALbreeding took a type at random for each pair of interacting genes. We also kept the epistatic pairs for each type of epistasis. These epistasis types were observed in studies of the inheritance of qualitative traits. Most simulation-based and empirical studies of quantitative traits fitting epistasis, including those involving genomic selection, GWAS, and QTL mapping, did not specify these epistasis types. Those with self-pollinated crops adjusted only the AxA genetic value. This shows that the significance of epistasis for complex traits remains a challenge. We further defined k = 1.5.

Both low- and high-LD populations were submitted to a recurrent genomic selection (GS) process over seven cycles, replicated 10 times. Considering 10 recent studies involving recurrent genomic selection, in five, the number of cycles was 5, 7, or 10. The others included 30 or 50 generations. Generation 1 was created by crossing the initial parents (generation 0). The progeny size was 10 individuals, maintained throughout all generations. In each selection cycle, we selected 400 females and 80 males based on the predicted additive genetic value. This corresponds to 20% and 4% of females and males selected, respectively. To control inbreeding, we instructed REALbreeding not to cross sibs. Then, from the selected females and males, the software defined five females per male, avoiding full-sib cross. The genomic selection assumed a training set of initial parents and 20% of the individuals in each generation, randomly chosen. Thus, we updated the prediction model in each generation, using historical data.

**Figure 1:**
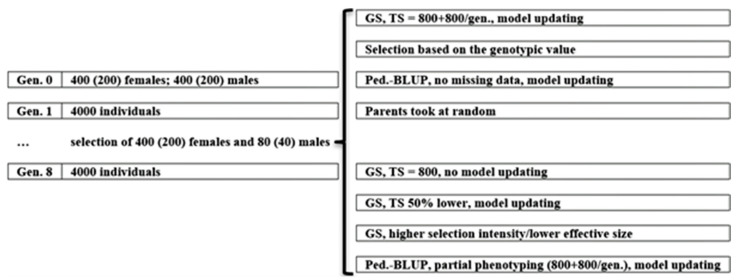
A graphical summary of the simulated scenarios (GS = genomic selection; TS = training set).

Under the additive–dominance model, three scenarios served as references: selection based on the true genotypic value (GV), which is provided by REALbreeding (because selection, the software does not provide additive and dominance genetic values), pedigree-based BLUP (pbB), and no selection (NS), in which we randomly provided 400 females and 80 males as parents to keep the same effective size (Figure 1). We also assumed additional scenarios for investigating the impact of decreasing training set size (lower cost) and proportion of selected individuals (higher selection intensity/lower effective population size). One additional scenario was genomic selection, assuming the initial parents as the training set (GS1; no model updating; lower phenotyping cost). A second additional scenario involved genomic selection with a training set that included initial parents and 10% of the individuals in each generation (GS2; a 50% decrease in the training set size, resulting in lower phenotyping costs). A third additional scenario involved selecting 200 females and 40 males, assuming a progeny size of 20 (GS3; a 50% decrease in the proportion of selected individuals/effective population size, resulting in higher selection intensity). Another additional scenario was pedigree-based BLUP assuming partial phenotyping—20% of the individuals in each generation (pbB1) (Figure 1). Partial phenotyping is better characterized for sex limited, difficult, or expensive to measure, or post-slaughtering traits, not as missing data. To investigate the impact of epistasis, the reference was the additive–dominance model.

### 2.3. Statistical Analysis

The simulated datasets were processed using the R package sommer 4.3.2 [21]. We fitted the additive–dominance and the additive–dominance with epistasis models. The complete model was $y=X\beta +Z_{1}a+Z_{2}d+Z_{3}aa+Z_{4}ad+Z_{5}dd+\varepsilon$, where Z is an incidence matrix and a, d, aa, ad, and dd are the vectors of additive, dominance, AxA, AxD, and DxD genetic values. We adopted the additive and the dominance genomic relationship matrices proposed by VanRaden [22] (first method) and Su et al. [23], respectively. We generated the AxA, AxD, and DxD genomic matrices by Hadamard products from the additive and dominance matrices. The AxD effects corresponded to the sum of AxD + DxA effects. To calculate the additive relationship matrices, we use the R package AGHmatrix 2.1.4 [24]. We computed the inbreeding coefficient from the diagonal elements of the genomic matrix and from the pedigree, also provided by REALbreeding, using the R package pedigreemm 0.3–3 [25]. We calculated the genotypic variance in each generation using the true genotypic values provided by REALbreeding. We estimated the additive value prediction accuracy by Pearson’s correlation between the predicted additive values and the true genotypic values. We calculated the genetic gains by the difference between the genotypic means of successive generations. To investigate the efficacy of both expected and genomic F to express the real population inbreeding level, we requested REALbreeding to save genotypes for genes (generations 1 to 8), using the ancestral alleles (generation 0). In the initial generation, each gene is identified by the individual’s index and identical by state (IBS) alleles in a homozygote are distinguished by an asterisk (for example, 1-1/1*-1, 1-1/2-1, and 2-1/2*-1). Then, we computed the realized F as the average number of alleles identical by descent, from counting over 38.5 K SNPs and 400 or 4000 individuals. We finally assessed the changes in allele frequencies resulting from selection using the gene genotypes.

## 3. Results

**Figure 2:**
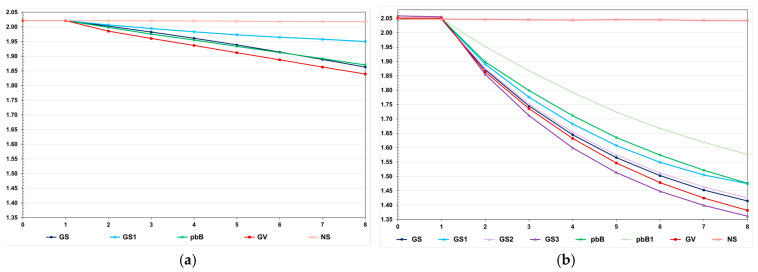
Average feed conversion ratio over generations in the low (**a**) and high (**b**) LD populations under the additive–dominance model, assuming GS—genomic selection with training set including initial parents and 20% of the individuals/generation, GS1—genomic selection with initial parents as training set, GS2—GS with 10% of phenotyped individuals, GS3—GS with higher selection intensity, GV—selection based on the true genotypic value, pbB—pedigree-based BLUP with phenotyping data from all individuals, pbB1—pedigree-based BLUP with the same training set as GS, and NS—no selection. The ranges for the standard deviation for the generation means were 0.02–0.03 (0.02 on average) and 0.02–0.11 (0.07 on average), for the low- and high-LD populations.

We observed a much higher selection efficacy in the high LD population, even for pedigree-based BLUP and selection based on the true genotypic value (Figure 2). For the genomic selection using parents and 20% of phenotyped individuals as the training set, the decrease in the FCR in the high LD population was four times higher than the decrease observed in the low LD population (−0.63 and −0.16). In the low LD population, the total genetic gain with genomic selection was 13.1% lower than the gain with selection based on the true genotypic value and 5.0% higher than the gain from pedigree-based BLUP. In the high LD population, the genomic selection efficacy was only 5.0% lower than the maximum value and 10.6% more efficient than pedigree-based BLUP. In both populations, using only the parents as a training set—but keeping the same proportion of selected individuals—decreased the genomic selection efficiency, especially in the low LD population (−8.9 and −55.0%). In the high LD population, increasing the genomic selection intensity increased the total genetic gain by 8.6%. Curiously, this selection procedure was 3.2% more efficient than selection based on the true genotypic value. By decreasing the costs of phenotyping by 50%, the genomic selection efficiency decreased by only 1.6%. Pedigree-based BLUP with partial phenotyping was the least efficient method. The decrease in total genetic gain was 17.8% compared to 100% phenotyping.

**Figure 3:**
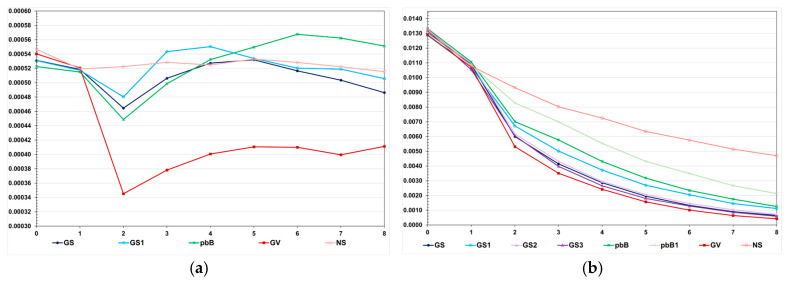
Average genotypic variance for feed conversion ratio over generations in the low (**a**) and high (**b**) LD populations, under the additive–dominance model, assuming GS—genomic selection with training set including initial parents and 20% of the individuals/generation, GS1—genomic selection with initial parents as training set, GS2—GS with 10% of phenotyped individuals, GS3—GS with higher selection intensity, GV—selection based on the true genotypic value, pbB—pedigree-based BLUP with phenotyping data from all individuals, pbB1—pedigree-based BLUP with the same training set as GS, and NS—no selection. The ranges for the standard deviation of the genotypic variances were 0.00001–0.00007 (0.00003 on average) and 0.0000–0.0018 (0.00039 on average), for the low- and high-LD populations.

**Figure 4:**
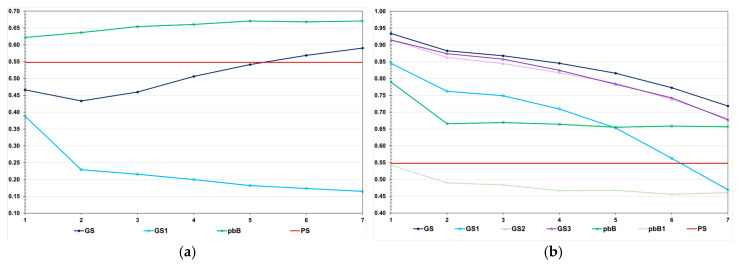
Average additive value prediction accuracy for feed conversion ratio over generations in the low (**a**) and high (**b**) LD populations, under the additive–dominance model, assuming GS—genomic selection with training set including initial parents and 20% of the individuals/generation, GS1—genomic selection with initial parents as training set, GS2—GS with 10% of phenotyped individuals, GS3—GS with higher selection intensity, pbB—pedigree-based BLUP with phenotyping data from all individuals, pbB1—pedigree-based BLUP with the same training set as GS, and PS—phenotypic selection. The ranges for the standard deviation for the prediction accuracies were 0.01–0.05 (0.03 on average) and 0.01–0.07 (0.02 on average), for the low- and high-LD populations.

The decreases in the genotypic variance were higher in the high LD population (Figure 3). Regardless of the genetic assessment procedure, the decreases in the high LD population ranged from 83.6 to 96.7%, perfectly proportional to the total genetic gain. The decreases in the low LD population ranged from 5.0 to 31.4%, but the correlation with the total genetic gain was 0.48. In the high LD population, we observed a significant decrease in the genotypic variance under no selection (64.7%). This occurred because the LD values were predominantly positive. We increased the effective population size to 800 and 2000 under no selection, and the decreases in the genotypic variance were the same—approximately 60%. In the low LD population, under no selection, the decrease in the genotypic variance due to the decrease in the LD was only 5.7%. The decrease in the genetic variability did not significantly affect the additive genetic prediction accuracy, except for genomic selection using only parents as the training set, regardless of the LD level (Figure 4).

In the low LD population, the prediction accuracies with genomic selection were lower than the accuracies for pedigree-based BLUP. The opposite occurred in the high LD population. In the high LD population, except for pedigree-based BLUP with partial phenotyping, the prediction accuracies were generally higher than the expected accuracy by phenotypic selection (0.55) (Figure 4). In this population, regardless of the selection process, we observed a decrease in the prediction accuracy, especially for genomic selection using parents as the training set. The decreases ranged from 15.0 to 44.5%. Important to emphasize, except for pedigree-based BLUP with partial phenotyping in the low LD population, the correlations between accuracies and absolute genetic gains were of high magnitude (0.87–0.94).

**Figure 5:**
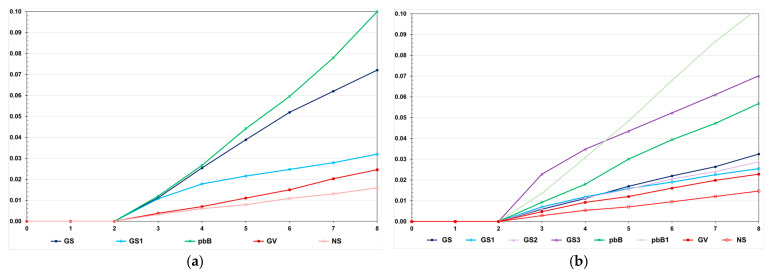
Average inbreeding coefficient over generations in the low (**a**) and high (**b**) LD populations, under the additive–dominance model, assuming GS—genomic selection with training set including initial parents and 20% of the individuals/generation, GS1—genomic selection with initial parents as training set, GS2—GS with 10% of phenotyped individuals, GS3—GS with 20% of phenotyped individuals and higher selection intensity, GV—selection based on the true genotypic value, pbB—pedigree-based BLUP with phenotyping data from all individuals, pbB1—pedigree-based BLUP with the same training set as GS, and NS—no selection. The ranges for the standard deviation for the inbreeding coefficients were 0.01–0.05 (0.03 on average) and 0.01–0.07 (0.03 on average), for the low- and high-LD populations.

Under no selection, but avoiding sib crosses, the inbreeding coefficient in the last generation was approximately 0.02, regardless of the LD level (Figure 5). Applying selection, the F-value in the last generation ranged from approximately 0.02 to 0.10, regardless of the LD level. The higher values were achieved by pedigree-based BLUP selection. Selection based on the true genotypic value minimized inbreeding in both populations. Genomic selection assuming parents and 20% of phenotyped individuals as a training set increased the inbreeding coefficient from 0.01 to 0.03 and 0.07, in the high- and low-LD population, respectively. Increasing the selection intensity increased the inbreeding coefficient from 0.02 to 0.07. Changes in the training set did not affect the inbreeding level.

**Figure 6:**
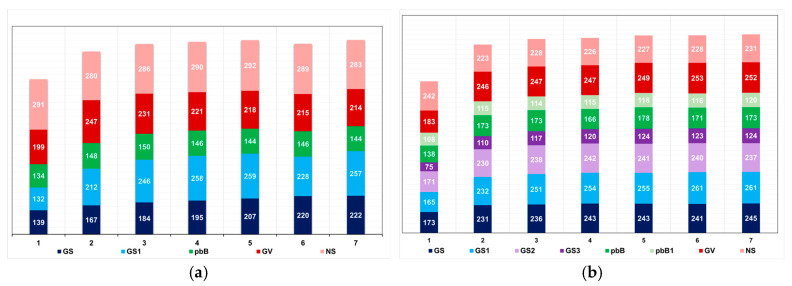
Average number of progenies with selected individuals over generations in the low (**a**) and high (**b**) LD populations, under the additive–dominance model, assuming GS—genomic selection with training set including initial parents and 20% of the individuals/generation, GS1—genomic selection with initial parents as training set, GS2—GS with 10% of phenotyped individuals, GS3—GS with 20% of phenotyped individuals and higher selection intensity, GV—selection based on the true genotypic value, pbB—pedigree-based BLUP with phenotyping data from all individuals, pbB1—pedigree-based BLUP with the same training set as GS, and NS—no selection. The ranges for the standard deviation of the number of progenies were 4–80 (9.5 on average) and 3–23 (7.6 on average) for the low- and high-LD populations.

**Figure 7:**
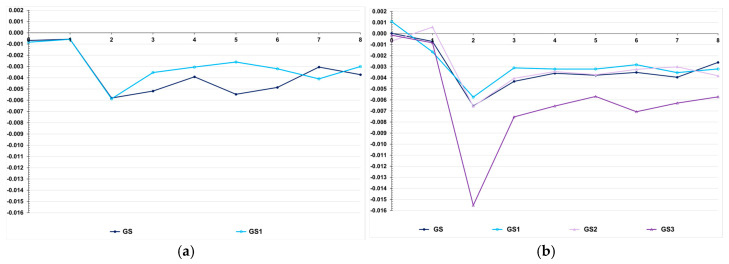
Average genomic inbreeding coefficient over generations in the low (**a**) and high (**b**) LD populations, under the additive–dominance model, assuming GS—genomic selection with training set including initial parents and 20% of the individuals/generation, GS1—genomic selection with initial parents as training set, GS2—GS with 10% of phenotyped individuals, and GS3—GS with 20% of phenotyped individuals and higher selection intensity. The ranges for the standard deviation for the genomic inbreeding coefficients were 0.02–0.08 (0.04 on average) and 0.04–0.09 (0.06 on average), for the low- and high-LD populations.

The most important factor impacting the level of inbreeding was the number of selected progeny (or the number of individuals selected by progeny) (Figure 6). In the low LD populations, the methods that minimized and maximized the average number of selected progenies were pedigree-based BLUP (145 progenies, F = 0.10) and genomic selection, including only parents in the training set (227 progenies, F = 0.03), respectively. In the high LD population, the processes that minimized the number of selected progenies were genomic selection under high selection intensity (113 progenies, F = 0.07) and pedigree-based BLUP with partial phenotyping (115 progenies, F = 0.10). The process that maximized the number of selected progenies was genomic selection, including only parents in the training set (240 progenies, F = 0.02). The genomic inbreeding coefficient over generations was close to zero (Figure 7). The correlations between the probabilistic and genomic inbreeding coefficients were 0.57 and −0.02 in the low LD population. In the high LD population, the range was from −0.26 to 0.73. Irrespective of the population, the correlations between the genomic F and the number of selected progenies ranged from −0.37 to −0.73.

**Figure 8:**
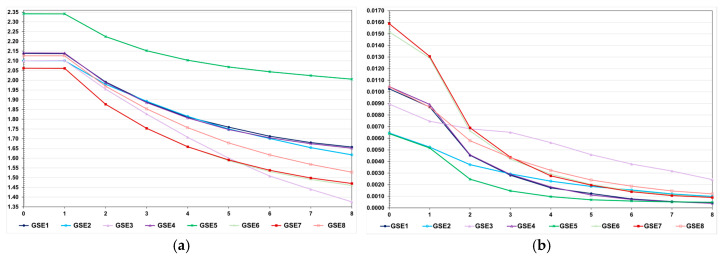
Average feed conversion ratio (**a**) and genotypic variance (**b**) over generations in the high LD population, assuming seven types of epistasis and an admixture of types (E1 = complementary; E2 = duplicate; E3 = dominant; E4 = recessive; E5 = dominant and recessive; E6 = duplicate genes with cumulative effects; E7 = non-epistatic gene interaction; and E8 = all types), and GS—genomic selection with training set including initial parents and 10% of the individuals/generation. The ranges for the standard deviation for the generation means and genotypic variances were 0.02–0.13 (0.06 on average) and 0.000–0.001 (0.0003 on average), respectively.

Assuming 50% of interacting genes, except for dominant epistasis, the efficacy of genomic selection was 4.1 to 46.2% lower than the efficacy under no epistasis (Figure 8). This negative effect is impressive considering that the sum of the epistatic genetic variances corresponds to approximately 1 to 3.5% of the genotypic variance. Assuming dominant epistasis, the genomic selection efficacy was 16.4% higher than the efficacy under no epistasis. Regardless of the epistasis type, the genotypic variance significantly decreased after seven selection cycles (−67 to −96%) (Figure 8). As observed for the additive–dominance model, this decrease is attributable to selection and recombination, because of predominantly positive LD values. Irrespective of the epistasis type, the decrease under no selection ranged from approximately 56 to 65% (60% on average).

**Figure 9:**
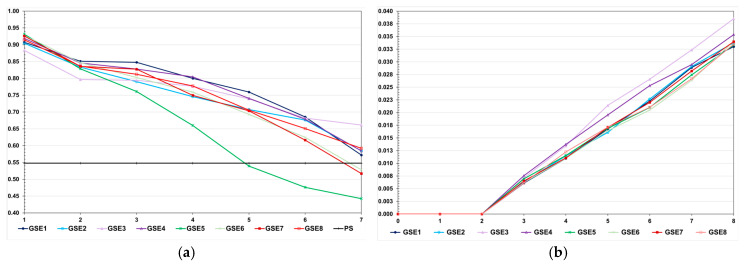
Average additive value prediction accuracy for feed conversion ratio (**a**) and inbreeding coefficient (**b**) over generations in the high LD population, assuming seven types of epistasis and an admixture of types (E1 = complementary; E2 = duplicate; E3 = dominant; E4 = recessive; E5 = dominant and recessive; E6 = duplicate genes with cumulative effects; E7 = non-epistatic gene interaction; and E8 = all types), GS—genomic selection with training set including initial parents and 10% of the individuals/generation, and PS—phenotypic selection. The ranges for the standard deviation for the prediction accuracies and inbreeding coefficients were 0.01–0.06 (0.03 on average) and 0.021–0.025 (0.023 on average), respectively.

**Figure 10:**
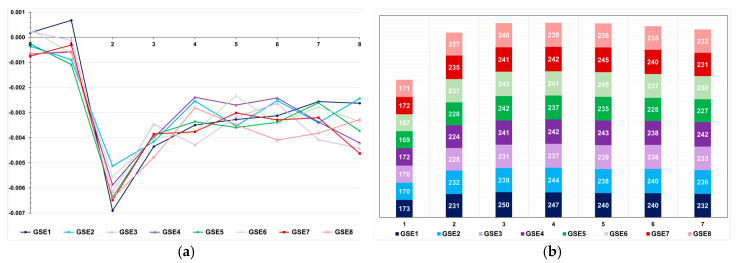
Average genomic inbreeding coefficient (**a**) and number of progenies with selected individuals over generations in the high LD population (**b**), assuming seven types of epistasis and an admixture of types (E1 = complementary; E2 = duplicate; E3 = dominant; E4 = recessive; E5 = dominant and recessive; E6 = duplicate genes with cumulative effects; E7 = non-epistatic gene interaction; and E8 = all types), and GS—genomic selection with training set including initial parents and 10% of the individuals/generation. The ranges for the standard deviation for the genomic inbreeding coefficients and number of progenies were 0.04–0.09 (0.06 on average) and 5–13 (9 on average), respectively.

Compared to the genotypic variance assuming additive–dominance model (Figure 3, high LD), the genotypic variance in generation 0 under epistasis was higher with dominant, duplicate genes with cumulative effects, and non-epistatic gene interaction (7.1 to 23.3%) and lower with the other types (−10.4 to −68.8%) (Figure 8). Interestingly, under dominant epistasis, the genetic gain was maximized and the decrease in the genotypic variance was minimized. For most epistasis types, the breeding value prediction accuracies were higher than the accuracy of phenotypic selection (Figure 9). The inbreeding coefficient in the last generation ranged from 0.03 to 0.04, values comparable to those observed for genomic selection under no epistasis (0.03) (Figure 9). As also observed under no epistasis, the genomic F was close to zero (Figure 10). Interestingly, the numbers of selected progenies/generation are very similar for all epistasis types and comparable to the numbers observed under no epistasis (Figure 10).

**Figure 11:**
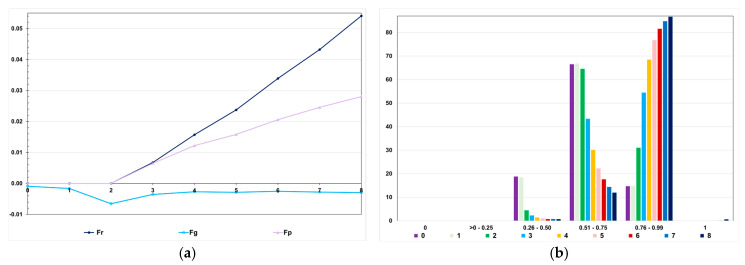
Average expected (p), genomic (g), and realized (r) inbreeding coefficients (**a**) and distribution of frequencies for the favorable alleles over generations in the high LD population (**b**), under the additive–dominance model, assuming genomic selection with a training set including initial parents and 10% of the individuals/generation.

We observed, assuming genomic selection with a training set including initial parents and 10% of the individuals/generation, that the realized F showed an almost perfect correlation with the expected F (0.996; generations 3 to 8) and a positive intermediate correlation with the genomic F (0.435) (Figure 11). However, the realized F (Fr) was higher than the expected F (Fp) (Fr = −0.01 + 2.19Fp; r2 = 0.99). The difference was proportional to the number of selection cycles. The analysis of the favorable allele frequency changes over the selection cycles, assuming the same genomic selection procedure, showed changes from −0.37 to 0.55 (0.27 on average). In the generation 0, no favorable allele was lost or fixed. The range for the favorable allele frequency in the parents was 0.26–0.97 (0.61 on average). After seven selection cycles, the range shifted to 0.29–1.00 (0.88 on average), but only five favorable alleles were fixed (Figure 11).

## 4. Discussion

Our main contributions on the efficiency of recurrent genomic selection in panmictic populations are that genomic selection efficacy, expressed as realized total genetic gain, is proportional to the population LD level and that, except for dominant epistasis, epistasis negatively impacts its efficacy, compared to the efficacy under additive–dominance model. The genomic selection using parents and 20% of phenotyped individuals in each generation as the training set provided a total genetic gain in the high LD population that was four times higher than the gain achieved in the low LD population. In the study of Thomasen, Liu, and Sorensen [8], genomic selection in the large high LD population provided a genetic gain 9.1% higher than the genetic gains in the large low LD populations. Our results demonstrate that the additive genomic relationship matrix effectively expresses the genotypic similarities between individuals, due to their genetic relationship or identity by descent for genes, if relatives, and genomic similarity or IBS for SNPs, which depends on the LD between genes and SNPs.

Assuming 50% of interacting genes, the efficacy of genomic selection was 4.1 to 46.2% lower than the efficacy under no epistasis, excepting dominant epistasis. Wientjes, Bijma, Calus, Zwaan, Vitezica, and van den Heuvel [7] also observed that epistasis negatively affected the accumulated genetic gains. The decrease compared to the additive model was approximately 27% for GBLUP, but only approximately 1% when dominance was included. They also observed lower changes in the allele frequencies over 50 generations under epistasis, relative to the additive model. Compared with the additive value prediction accuracy under no epistasis, in 91% of our cases (seven generations x eight epistasis scenarios), the additive value prediction accuracy was lower under digenic epistasis, even for dominant epistasis. The additive value prediction accuracy under epistasis ranged from −35.5 to 1.8% relative to the accuracies assuming no epistasis.

Why did only dominant epistasis genomic selection provide efficacy higher than the efficacy assuming the additive–dominance model remains unclear for us. We can associate it with keeping the highest genotypic variance since generation 2. This can justify the maximum accuracy in the last two cycles. Important studies on the impact of epistasis on selection did not specify digenic epistasis types. Assuming the infinitesimal model, the theoretical results showed that the effect of epistasis on the population mean change due to selection may be modest and the response slower, because epistatic variance represents only a small part of the genotypic variance [26,27]. In contrast, Hansen [28] showed that the impact is negligible in the short term but may increase with time.

After seven selection cycles based on genomic selection using parents and 10% of phenotyped individuals, the accumulated genetic gains for FCR in the high LD population were −30.4 and −25.4%, on average, assuming no epistasis and digenic epistasis, respectively. Thus, the absolute average rates of genetic gain were 4.3 and 3.6%, respectively. The maximum average rates of genetic gains observed in previous investigations using simulated data ranged from 0.5 to 2.6%, proportional to the trait heritability [4,5,7,8,29]. It is essential to note that this genomic selection procedure, which minimizes costs, resulted in a non-significant increase in both expected and genomic inbreeding coefficients. The low F values observed after seven genomic selection cycles—0.03 to 0.07—are mainly due to the avoidance of sib crosses. The genomic F values were 10 times lower. We also observed that, except for genomic selection under high selection intensity, the genomic selection procedures provided high numbers of selected progenies (low average number of selected full-sibs) in both additive–dominance and additive–dominance with epistasis models, regardless of the epistasis type.

Zhao, Zhang, Wang, Akdemir, Garrick, He, and Wang [4] concluded that genomic mating effectively controls the rates of inbreeding accumulation in the population. Assuming a heritability of 0.30, the inbreeding coefficient for the genomic matings ranged from 0.07 to 0.10 after five generations. Zheng, Zhang, Wang, Niu, Wu, Wang, Gao, Li, and Xu [5] observed inbreeding coefficients in the range 0.05–0.06 after seven cycles, higher for GBLUP and BayesA. In an American Angus cattle population under genomic selection over 15 years, Lozada-Soto, Maltecca, Lu, Miller, Cole and Tiezzi [12] observed an increase in the inbreeding coefficient from approximately 0.04 to 0.06. The genomic F was much higher and increased from approximately 0.25 to 0.28. Makanjuola, Miglior, Abdalla, Maltecca, Schenkel, and Baes [14] also observed higher average genomic inbreeding coefficients in Holstein and Jersey cattle populations (0.31 and 0.43), relative to the average pedigree-based values (0.08 and 0.07). Over 18 years of selection, including genomic selection, the pedigree-based F increased from approximately 0.04 to 0.08–0.09, and the genomic F increased from 0.20 to 0.33 and from 0.41 to 0.43, in both populations. Low to intermediate negative and positive correlations between the expected and genomic F (−0.44 to 0.63) have been observed in several studies [12,30,31,32,33].

Regarding the impact of model updating, training set size, and selection intensity, our results agree with those obtained in studies based on simulated and empirical datasets. We observed that genomic selection requires model updating to achieve higher genetic gains. This is necessary because selection changes gene frequencies and, consequently, the LD between genes and SNPs, and recombination also alters the LD for genes and SNPs. Li, Zhang, Liu, and Chen [9] observed that genotyping 40–50% of each litter in the previous three to four generations can provide similar selection efficacy, assuming 100% of genotyping. A 50% decrease in phenotyping cost resulted in a 1.5% decrease in genomic selection efficacy, with no significant impact on additive value prediction accuracy (3.4% lower on average), genotypic variance (a lower decrease), and inbreeding level (12% lower). Obsteter et al. [34] concluded that, in dairy cattle breeding, the genetic gain increased by increasing genotyping, despite reduced phenotyping. By increasing the selection intensity (decreasing by 50% the number of males and females selected or the effective population size), the total genetic gain increased 9.6%. Interestingly, the additive value prediction accuracy was 2.9% lower on average, the genotypic variance in the last generation was 10.2% lower, and the inbreeding coefficient was 75% higher, but the magnitude was not high (0.06).

Regarding the significance of the expected additive relationship matrix, pedigree-based BLUP can only be as efficient as genomic selection in populations with low LD levels. Additionally, the genetic assessment for sex limited, difficult, or expensive to measure, or post-slaughtering traits is also more efficient by genomic selection than by pedigree-based BLUP. When there is a considerable proportion of non-measured individuals, pedigree-based BLUP is much less efficient than genomic selection (−26%) and leads to a much higher level of inbreeding (three times higher). The higher inbreeding results from a minimization of the number of selected progenies (maximization of the average number of full sibs selected). Probably because they simulated low LD dairy cattle populations, Seno et al. [35] did not observe differences between pedigree- and genomic-based selection, regarding genetic gains and inbreeding levels. Using a simulated livestock population, Wientjes, Bijma, Calus, Zwaan, Vitezica, and van den Heuvel [7] concluded that genomic selection outperformed pedigree selection in terms of long-term genetic gain. Both approaches were equivalent in decreasing the genotypic variance, and the decrease in heterozygosity was slightly lower for pedigree than for genomic selection. However, the relationship information has not been disregarded in animal breeding. By combining genomic and historical genetic relationship information, ssGBLUP became the most important method for genetic evaluations currently employed [36]. In the simulation-based studies of Buttgen et al. [37] and Pocrnic et al. [38], different long-term genotyping and selection strategies in laying hen breeding programs were assessed, including pedigree-based BLUP. Regardless of the generation interval, the rate of genetic progress was higher using genomic selection. In general, the rate of inbreeding was also higher with genomic selection.

## 5. Conclusions

The genomic selection efficacy, expressed as realized total genetic gain, is proportional to the LD level and genotypic variance. Only in low LD populations can pedigree-based BLUP be as efficient as genomic selection. Genomic selection requires model updating to achieve a higher efficacy. The training set size required by genomic selection can be as low as 10%/generation. Under this low-cost scenario, the genomic selection efficacy was only 5.5% lower than the maximum efficacy. There is no difference between genetic evaluation methods regarding the decrease in the genotypic variance. In general, additive value prediction accuracies and genetic gains were highly correlated. The accumulated inbreeding level under genomic selection was not high (3–7%) due to the avoidance of sib cross. The genomic inbreeding coefficient over generations was close to zero, irrespective of the genetic model. The realized F was almost perfectly correlated with the expected value, but showed a higher magnitude (4 to 93%), which was proportional to the selection cycle number. Except for dominant epistasis, the efficacy of genomic selection under epistasis was 4.1 to 46.2% lower than the efficacy assuming no epistasis. We emphasize, however, that genomic selection is efficient for complex traits affected by epistasis and that the inbreeding coefficient computed from the diagonal of the genomic additive relationship matrix varied randomly around zero, indicating no inbreeding. In contrast, the F achieved 7%, higher than the expected value.

## Data Availability

The dataset is available at https://doi.org/10.6084/m9.figshare.26866762.v1 (accessed on 6 September 2024), https://doi.org/10.6084/m9.figshare.26947903.v2 (accessed on 6 September 2024), https://doi.org/10.6084/m9.figshare.26951263.v1 (accessed on 6 September 2024), and https://doi.org/10.6084/m9.figshare.26952103.v1 (accessed on 6 September 2024).

## Abbreviations

The following abbreviations are used in this manuscript:

F	Inbreeding coefficient
GS	Genomic selection
GV	Selection based on the true genotypic value
LD	Linkage disequilibrium
NS	No selection
pbB	Pedigree-based BLUP
